# Synthetic Studies on Bioactive Natural Polyketides: Intramolecular Nitrile Oxide-Olefin Cycloaddition Approach for Construction of a Macrolactone Skeleton of Macrosphelide B

**DOI:** 10.3390/molecules16064850

**Published:** 2011-06-10

**Authors:** Seung-Mann Paek, Young-Ger Suh

**Affiliations:** 1 College of Pharmacy, the Institute of Health Sciences, Gyeongsang National University, Jinju daero, Jinju, Gyeongnam, 660-751, Korea; Email: million@gnu.ac.kr (S-M.P.); 2 College of Pharmacy, Seoul National University, Gwanak-ro 599, Gwanak-gu, Seoul 151-742, Korea

**Keywords:** intramolecular nitrile oxide cycloaddition, macrosphelide, polyketide, total synthesis, phase transfer catalyst

## Abstract

Studies on the synthesis of macrosphelide B via an intramolecular nitrile oxide-olefin cycloaddition (INOC) is described. In particular, an asymmetric INOC approach using phase transfer catalysts seems to be a potentially efficient and versatile procedure for the construction of the macrolactone skeleton of macrosphelide B in terms of facial selectivity. Our preliminary and unprecedented stereoselective procedure is anticipated to be usefully applied through further studies for the synthesis of the macrosphelide family.

## 1. Introduction

**Scheme 1:**
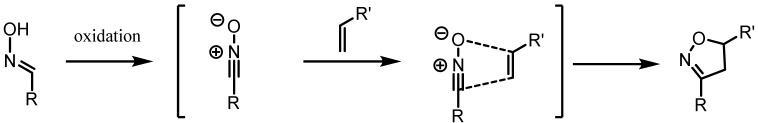
Nitrile oxide cycloaddition.

Macrocyclic polyketides are considered promising natural resources for development of biologically active molecules, including new medicines [1], although many of them consist of a synthetically labile framework. In particular, the macrocyclic ring-closure reaction has been frequently a formidable problem, due to the unfavorable enthalpy or entropy [2]. Thus, a number of synthetic methods such as a mixed anhydride mediated macrolactonization [3], Pd(0)-catalyzed cross coupling [4] and ring-closing metathesis [5,6] have been developed to overcome the intrinsic cyclization problem. Recently, the so-called intramolecular nitrile oxide-olefin cycloaddition (INOC, Scheme 1) approach has turned out as an alternative procedure for the requisite cyclization. [7,8]

INOC [9,10] can serve as an excellent pathway for the generation of the new chiralities along with the ring-closure operation [11], because it proceeds through a concerted mechanism. Indeed, a large number of INOC approaches have been reported as good procedures for the synthesis of medium-sized cyclic skeletons from a viewpoint of efficient chiral transfer and ring-closure [12]. However, few macrocyclic ring-closure reactions employing INOC have been reported.

**Scheme 2:**
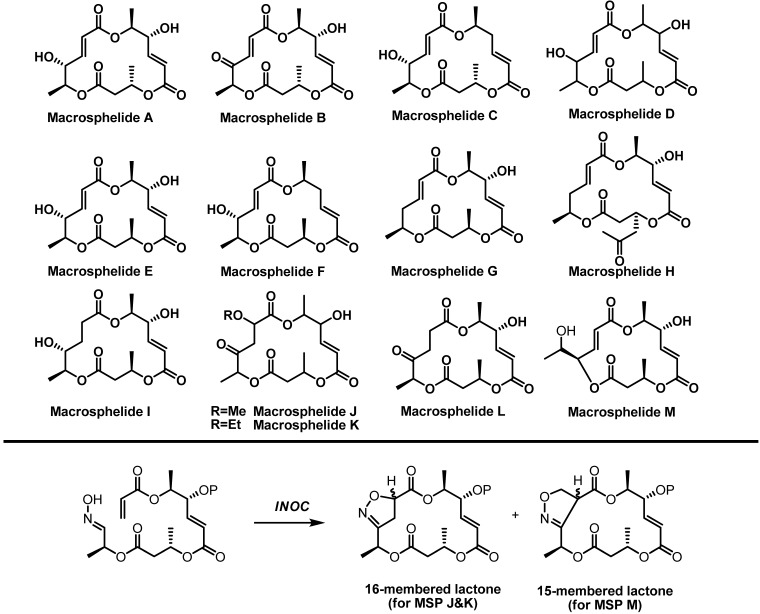
Structures of macrosphelides and INOC application for their syntheses.

Recently, we reported an asymmetric total synthesis of macrosphelides (MSP) [13,14,15], which have been attracting interest from both biologists and chemists because of their potent anticancer, apoptotic, and immunosuppressant activities as well as unique structural features [16,17,18,19,20,21]. In contrast to the synthesis of macrosphelide A (MSPA) via Yamaguchi macrolactonization [3], synthesis of macrosphelide B (MSPB) employed INOC for construction of the macrocyclic skeleton, which avoided the undesired side reactions, such as epimerization or formation of the geometric isomer [13]. With this early success, we extended our work to a mechanistic study of INOC [14] and an investigation on its regio- and stereochemical control using chiral catalysts. In addition, we anticipated expanding its synthetic utilities to the structural variation of MSPs for further biological improvement [22,23,24]. The asymmetric INOC could be effectively applied to the asymmetric synthesis of MSPJ and MSPK [15]. Moreover, it would provide the regioselective ring formation for construction of the uncommon 15-membered macrolide framework of MSPM (Scheme 2). In this article, we describe our recent effort for new variants of the regio- and stereoselective INOC, utilized for MSPB synthesis.

## 2. Results and Discussion

**Scheme 3:**
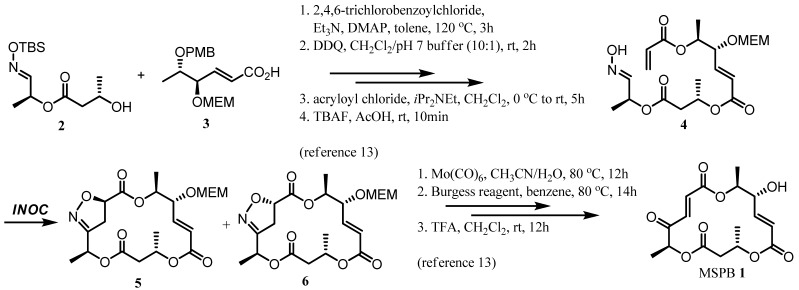
Synthetic route to MSPB via INOC.

Investigation on stereochemical control of INOC employed for the synthesis of MSPB (**1**) commenced with examination of various metal catalysts and the corresponding ligands. Aldoxime **4** is a precursor of the key intermediates **5** and **6** (Scheme 3) which can be efficiently transformed into MSPB (**1**) via a synthetic route developed by us [13]. It was also envisioned that successful stereocontrol of INOC would provide an invaluable transformation for MSP syntheses.

**Scheme 4:**
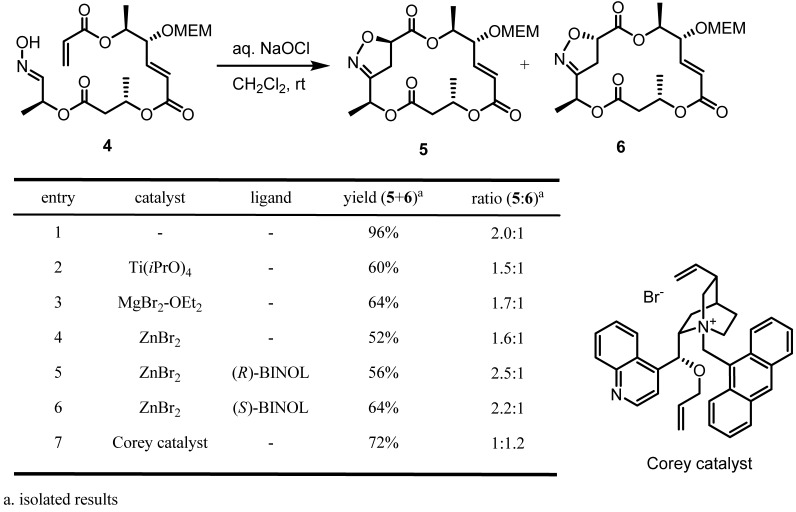
INOC of aldoxime **4** in the presence of various catalysts.

Our initial study on stereocontrol of INOC by metal catalysts for the synthesis of MSPB is summarized in Scheme 4. As reported earlier, [13] a simple addition of aqueous NaOCl to oxime precursor **4** afforded a mixture of **5** and **6** in a good yield and with moderate facial selectivity. [25] Considering the dramatic increase of diastereo- and enantioselectivity by various metal cations with chiral ligands in nitrile oxide cycloaddition [11], we have also examined metal cations (entries 2-4). However, no significant improvement of diastereomeric ratio with decrease of chemical yields was observed. Addition of chiral ligands was not beneficial either, although (*R*)-BINOL induced a slight increase of facial selectivity. Interestingly, addition of Corey phase-transfer catalyst [26] under cycloaddition conditions afforded an inversion of facial selectivity to produce isoxazoline **6** as a major product although the moderate selectivity still hampered practical use of this condition. To the best of our knowledge, PTC-induced reverse facial selectivity in the INOC-mediated macro ring-closure reaction has not been reported yet.

**Scheme 5:**
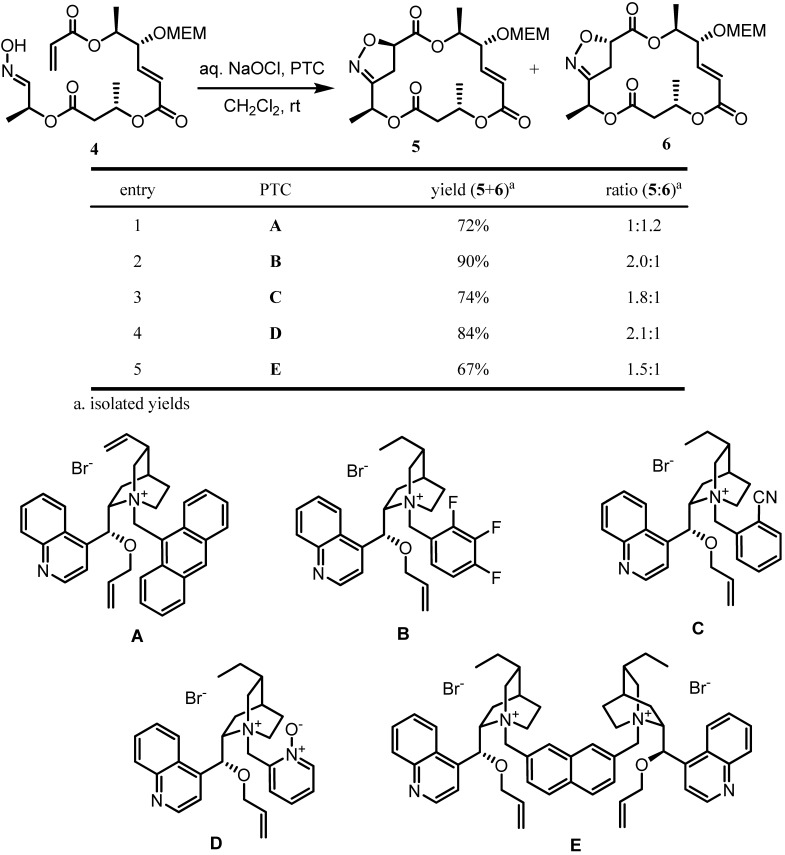
INOC of aldoxime **4**in the presence of various PTCs.

Inspired with the interesting result from the PTC-assisted INOC, we intensively examined other PTC catalysts [27] for improved selectivity, as summarized in Scheme 5. Although other PTCs seemed not to influence much the facial selectivity of INOC, potential chiral induction by PTC in INOC was observed. In most cases, high chemical yields were observed with a variety of stereoselectivities. The reason for the change of facial selectivity is not clear at present. However, it is likely due to an effect of the coexistence of water and CH_2_Cl_2_ as solvents.

**Scheme 6:**
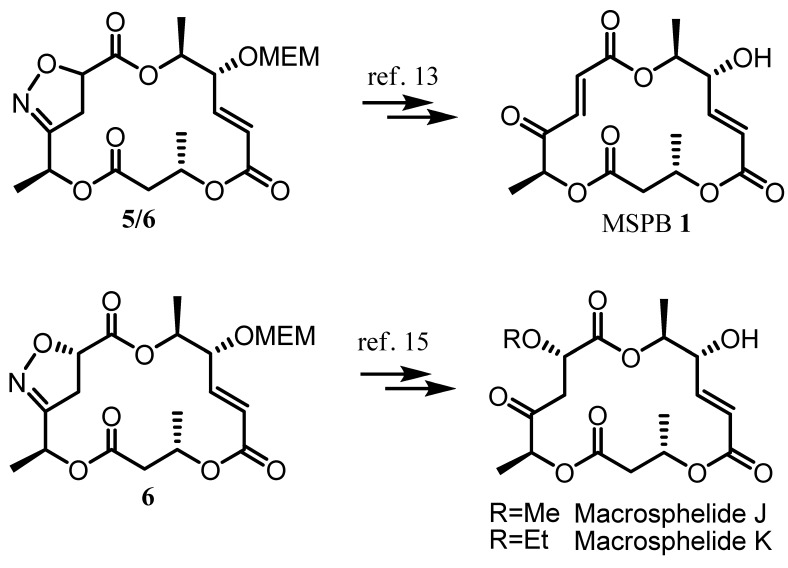
Conversion of isoxazoline intermediates to macrosphelides.

Ioxazolines **5** and **6** could be efficiently transformed to MSPB, MSPJ and MSPK through appropriate synthetic sequences as shown in Scheme 6 [13,28].

## 3. Experimental

### 3.1. General

Unless noted otherwise, all starting materials and reagents were obtained from commercial suppliers and were used without further purification. Tetrahydrofuran and Et_2_O were distilled from sodium benzophenone ketyl. Dichloromethane, triethylamine, acetonitrile and pyridine were freshly distilled from calcium hydride. All solvents used for routine isolation of products and chromatography were reagent grade and glass distilled. Reaction flasks were dried at 100 °C. Air and moisture sensitive reactions were performed under argon atmosphere. Flash column chromatography was performed using silica gel 60 (230-400 mesh) with the indicated solvents. Thin*-*layer chromatography was performed using 0.25 mm silica gel plates. Optical rotations were measured on JASCO DIP 1000 digital polarimeter using 100 nm cell of 1~2 mL capacity. Chemical shifts are expressed in parts per million (ppm, *δ*) downfield from tetramethylsilane and are referenced to the deuterated solvent (CHCl_3_). ^1^H-NMR data (on Bruker JEOL LNM-LA 300, JEOL JNM-GCX 400 and AMX-500 MHz systems) are reported in the order of chemical shift, multiplicity (s, singlet; d, doublet; t, triplet; q, quartet; m, multiplet and/or multiple resonance), number of protons, and coupling constant in Hertz (Hz).

*(1S)-2-([tert-Butyl(dimethyl)silyl]oxyimino)-1-methylethyl (3S)-3-[(4-methoxybenzyl)oxy] butanoate* (**2**). To a solution of (*S*)-2-(4-methoxybenzyloxy)propanal O-*tert*-butyldimethylsilyl oxime [13] (2.0 g, 6.1 mmol) in CH_2_Cl_2_/phosphate buffer solution (10:1, 88 mL, pH 7.0) at ambient temperature was added DDQ (2.1 g, 9.2 mmol). The reaction mixture was stirred for 1 h, diluted with CH_2_Cl_2_ and filtered under reduced pressure. The organic layer was washed with H_2_O and aqueous layer was extracted with Et_2_O. The combined organic layers were dried over MgSO_4_ and concentrated *in vacuo*. The residue was purified by flash column chromatography on silica gel (Et_2_O-*n-*hexane = 1:3) to afford 1.8 g of alcohol **16** and *p*-anisaldehyde as the inseparable mixture. This crude mixture was thus used for next step without further purification. To a solution of crude mixture (1.8 g) and (*S*)-3-(4-methoxybenzyloxy)butanoic acid [13] (1.3 g, 6.1 mmol) in CH_2_Cl_2_ (50 mL) at ambient temperature were added EDCI (1.1 g, 6.1 mmol) and DMAP (750 mg, 6.1 mmol). After stirring for 12 h, the reaction mixture was quenched with H_2_O, extracted with CH_2_Cl_2_ and aqueous layer was extracted with EtOAc. The combined organic layers were dried over MgSO_4_ and concentrated *in vacuo*. The residue was purified by flash column chromatography on silica gel (EtOAc-*n-*hexane = 1:9) to afford 1.7 g (70%) of (*S*)-[(*S*)-1-(*tert*-butyldimethylsilyloxyimino)propan-2-yl)] 3-hydroxybutanoate as a colorless oil: ^1^H-NMR (CDCl_3_, 300 MHz , *E/Z* mixture of aldoxime ethers): δ 7.33, 6.67 (d, 1H, *J* = 5.1 Hz), 7.08 (d, 2H, *J* = 8.6 Hz), 6.67 (d, 2H, *J* = 8.8 Hz), 5.74, 5.32 (dq, 1H, *J* = 5.1, 6.6 Hz), 4.30 (q, 2H, *J* = 10.9 Hz), 3.83 (m, 1H), 3.63 (s, 3H), 2.52 - 2.23 (m, 2H), 1.23 (d, 3H, *J* = 6.3 Hz), 1.08 (d, 3H, *J* = 6.0 Hz), 0.76 (s, 9H), 0.00 (m, 6H); ^13^C-NMR (CDCl_3_, 100 MHz, *E/Z* mixture of aldoxime ethers) δ 170.4, 159.1, 155.4, 153.3, 129.2, 113.7, 77.2, 71.6, 71.5, 70.6, 70.5, 67.9, 65.8, 55.2, 42.0 , 25.9 , 19.8, 18.1, 17.8, 16.9, -5.3, -5.4; IR (neat) ν _max_ 2932, 2857, 1741, 1613, 1513, 1464, 1373, 1298 cm^−1^; LRMS (FAB) *m/z* 409 (M^+^).

To a solution of the (*S*)-((*S*)-1-(*tert*-butyldimethylsilyloxyimino)propan-2-yl) 3-hydroxybutanoate (1.1 g, 2.7 mmol) in CH_2_Cl_2_/phosphate buffer solution (20:1, 32 mL, pH 7.0) at ambient temperature was added DDQ (670 mg, 2.9 mmol). The reaction mixture was stirred for 2 h and the reaction mixture was diluted with CH_2_Cl_2_, filtered under reduced pressure. The organic layer was washed with H_2_O, dried over MgSO_4_ and concentrated *in vacuo*. The residue was purified by flash column chromatography on silica gel (EtOAc-*n-*hexane = 1:5) to afford 650 mg (84%) of alcohol **2** as a colorless oil. ^1^H-NMR (CDCl_3_, 300 MHz , *E/Z* mixture of aldoxime ethers) δ 7.46, 6.84 (d, 1H, *J* = 4.9 Hz), 5.91, 5.48 (dq, 1H, *J* = 4.9, 6.7 Hz), 4.17 (bs, 1H), 2.96, 2.87 (d, 1H, *J* = 3.3 Hz), 2.51 - 2.36 (m, 2H), 1.39 (d, 3H, *J* = 6.7 Hz), 1.20 (d, 3H, *J* = 6.2 Hz), 0.89 (s, 9H), 0.14 (m, 6H); ^13^C-NMR (CDCl_3_, 100 MHz, *E/Z* mixture of aldoxime ethers) δ 171.9, 171.8, 153.0, 68.1, 65.9, 64.2, 64.1, 42.9, 42.7, 25.9, 22.4, 18.1, 18.0, 17.8, 17.0, -5.1, -5.3; IR (neat) ν _max_ 3435, 2933, 2858, 1738, 1465, 1375, 1253, 1175 cm^−1^; LRMS (FAB) *m/z* 290 (M + H^+^); HRMS (FAB) Calcd for C_13_H_28_NO_4_Si (M + H^+^): 290.1788, Found 290.1791.

*(E,4R,5S)-4-((2-methoxyethoxy)methoxy)-5-(4-methoxybenzyloxy)hex-2-enoic acid* (**3**). To a solution of (3R,4S,E)-4-(4-methoxybenzyloxy)-1-(4-methyl-2,6,7-trioxabicyclo[2.2.2]octan-1-yl)pent-1-en-3-ol [13] (87 mg, 0.25 mmol) in CH_2_Cl_2_ (5 mL) were added *i-*Pr_2_Net (130 μL, 0.75 mmol) and MEMCl (56 μL, 0.50 mmol) subsequently at 0 °C. After stirring of the mixture for 5 h at the ambient temperature, additional *i-*Pr_2_NEt (260 μL, 1.5 mmol) and MEMCl (112 μL, 1.0 mmol) were added subsequently at 0 °C. The reaction mixture was stirred at ambient temperature for 36 h and quenched with H_2_O. This mixture was extracted with CH_2_Cl_2_ and aqueous layer was extracted with EtOAc. The combined organic layers were dried over Na_2_SO_4_ and concentrated *in vacuo*. This crude mixture was used for next step without further purification. To a solution of MEM ether (190 mg, crude product) in THF (4 mL) and H_2_O (4 mL) was added 1*N* HCl (0.1 mL). The reaction mixture was stirred for 10 min at ambient temperature, and lithium hydroxide monohydrate (80 mg, 1.9 mmol) was added. After stirring for an additional 2 h, the reaction mixture was acidified with 1 *N* HCl (pH < 3) and extracted with EtOAc. The combined organic layers were washed with brine, dried over MgSO_4_ and concentrated *in vacuo*. The residue was purified by flash column chromatography on silica gel (CH_2_Cl_2_-MeOH = 15:1) to afford 76 mg (86%, for 2 steps) of acid **3** as a colorless oil: [α]_D_^20^ -39.3 (c 0.5, MeOH); ^1^H-NMR (CDCl_3_, 300 MHz) δ 7.17 (d, 2H, *J* = 8.6 Hz), 6.93 (dd, 1H, *J* = 5.9, 15.7 Hz), 6.79 (d, 2H, *J* = 8.6 Hz), 5.99 (dd, 1H, *J* = 1.5, 15.7 Hz), 4.69 (dd, 2H, *J* = 7.0, 19.1 Hz), 4.44 (s, 2H), 4.31 - 4.27 (m, 1H), 3.75 - 3.65 (m, 1H), 3.72 (s, 3H), 3.65 - 3.47 (m, 1H), 3.45 - 3.42 (m, 2H), 3.32 - 3.30(m, 1H), 3.30 (s, 3H), 1.12 (d, 3H, *J* = 6.4 Hz); ^13^C-NMR (CDCl_3_, 75 MHz) δ 170.6, 159.1, 147.8, 130.2, 129.2, 113.7, 94.2, 77.6, 76.0, 71.6, 70.8, 67.2, 58.9, 55.2, 15.6; IR (neat) ν _max_ 2935, 1717, 1613, 1513, 1459, 1248, 1175, 1099, 1035 cm^−1^; LRMS (FAB) *m/z* 355 (M + H^+^); HRMS (FAB) Calcd for C_18_H_27_O_7_ (M + H^+^): 355.1757, Found 355.1765.

*(4R,5S,E)-((S)-4-((S,E)-1-(hydroxyimino)propan-2-yloxy)-4-oxobutan-2-yl) 5-(acryloyloxy)-4-((2-methoxyethoxy)methoxy)hex-2-enoate* (**4**).To a solution of (2*E*,4*R*,5*S*)-((7*S*,11*S*)-2,2,3,3,7-pentamethyl-9-oxo-4,8-dioxa-5-aza-3-siladodec-5-en-11-yl) 5-(acryloyloxy)-4-((2-methoxyethoxy)methoxy)hex-2-enoate [13] (86 mg, 0.15 mmol) in THF (5 mL) at ambient temperature were added AcOH (2 drops) and TBAF (1.0 M in THF, 0.23 mL, 0.23 mmol). The reaction mixture was stirred for 10 min and quenched with H_2_O. The mixture was extracted with EtOAc and the combined organic layers were dried over MgSO_4_ and concentrated *in vacuo*. The residue was purified by flash column chromatography on silica gel (EtOAc-*n-*hexane = 1:1) to afford 69 mg (99%) of acryloyl oxime **4** as a colorless oil: ^1^H-NMR (CDCl_3_, 300 MHz, *E/Z* mixture of aldoximes) δ 7.98 (s, 1H), 7.33 (d, 1H, *J* = 5.6 Hz), 6.71 (dd, 1H, *J* = 6.0, 15.7 Hz), 6.36 (dd, 1H, *J* = 1.2, 17.2 Hz), 6.09 - 6.00 (m, 1H), 5.97 (dd, 1H, *J* = 1.2, 15.7 Hz), 5.79 (dd, 1H, *J* = 1.4, 10.4 Hz), 5.40 - 5.36 (m, 1H), 5.27 - 5.23 (m, 1H), 5.08 - 5.04 (m, 1H), 4.67 (dd, 2H, *J* = 6.9, 17.7 Hz), 4.37 - 4.35 (m, 2H), 3.74 - 3.68 (m, 1H), 3.61 - 3.54 (m, 1H), 3.47 - 3.44 (m, 2H), 3.31 (s, 3H), 2.67 - 2.46 (m, 2H), 1.32 (d, 3H, *J* = 6.5 Hz), 1.28 (d, 3H, *J* = 6.2 Hz), 1.18 (d, 3H, *J* = 6.6 Hz), 0.89 (s, 9H), 0.12 (s, 6H); ^13^C-NMR (CDCl_3_, 75 MHz, *E/Z* mixture of aldoximes) δ 169.2, 164.8, 149.6, 143.3, 131.3, 128.3, 124.1, 93.8, 77.2, 76.3, 71.6, 67.9, 67.2, 59.0, 41.0, 19.9, 17.9, 14.5; IR (neat) ν _max_ 3395, 2933, 1725, 1454, 1407, 1272, 1193, 1055 cm^−1^; LRMS (FAB) *m/z* 446 (M + H^+^); HRMS (FAB) Calcd for C_20_H_32_NO_10_ (M + H^+^): 446.2026, Found 446.2028.

### 3.2. Representative INOC Procedure

To a solution of oxime (2.5 mg, 5.61 μmol) and Corey catalyst **A** (4.0 mg, 6.60 μmol) in CH_2_Cl_2_ (1.0 mL) was added aqueous NaOCl (0.05 mL, c.a. 10%) at ambient temperature. The reaction mixture was stirred for 1 h at the same temperature and diluted with H_2_O. The aqueous layer was extracted with CH_2_Cl_2_ and the combined organic layers were dried over MgSO_4_ and concentrated *in vacuo*. The residue was purified by flash column chromatography on silica gel (EtOAc:*n*-hexane = 2:3 to 2:1) to afford 0.8 mg of isoxazoline **5** and 1.0 mg of isoxazoline **6** as a pale yellow oil, respectively.

(2*S*,6*S*,11*R*,12*S*,15*R*)-*11-[(2-methoxyethoxy)methoxy]-2,6,12-trimethyl-3,7,13,16-tetraoxa-17-aza-bicyclo*[13.2.1]*octadeca-1(17),9-diene-4,8,14-trione* (**5**). [α]_D_^20^ -107.5 (c 0.8, CHCl_3_); ^1^H-NMR (CDCl_3_, 400 MHz) δ 6.68 (dd, 1H, *J* = 4.7, 15.7 Hz), 6.00 (d, 1H, *J* = 15.7 Hz), 5.47 (q, 1H, *J* = 6.5 Hz), 5.33 - 5.30 (m, 1H), 5.00 (dd, 1H, *J* = 7.5, 11.7 Hz), 4.89 (dq, 1H, *J* = 4.0, 6.4 Hz), 4.69 (dd, 2H, *J* = 7.0, 13.2 Hz), 4.21 (t, 1H, *J* = 8.0 Hz), 3.78 - 3.73 (m, 1H), 3.64 - 3.59 (m, 1H), 3.55 - 3.51 (m, 2H), 3.37 (s, 3H), 3.22 (dd, 1H, *J* = 11.9, 17.6 Hz), 3.07 (dd, 1H, *J* = 7.5, 17.6 Hz), 2.69 - 2.59 (m, 2H), 1.56 (d, 3H, *J* = 6.6 Hz), 1.42 (d, 3H, *J* = 6.2 Hz), 1.33 (d, 3H, *J* = 6.3 Hz); ^13^C-NMR (CDCl_3_, 100 MHz) δ 170.3, 169.1, 164.5, 157.4, 144.5, 125.1, 93.6, 78.1, 77.2, 77.1, 72.3, 71.5, 67.9, 67.4, 65.9, 59.0, 40.6, 40.4, 19.7, 17.4; IR (neat) ν _max_ 2924, 1739, 1455, 1371, 1272, 1187, 1051, 852 cm^−1^; LRMS (EI) *m/z* 443 (M + Na^+^); HRMS (FAB) Calcd for C_20_H_30_NO_10_ (M+H^+^): 444.1870, Found 444.1881.

*(2S,6S,11R,12S,15S)-11-[(2-methoxyethoxy)methoxy]-2,6,12-trimethyl-3,7,13,16-tetraoxa-17-azabicyclo*[13.2.1]*octadeca-1(17),9-diene-4,8,14-trione* (**6**). [α]_D_^20^ -55.1 (c 1.16, CHCl_3_); ^1^H-NMR (CDCl_3_, 300 MHz) δ 6.67 (dd, 1H, *J* = 7.8, 15.9 Hz), 5.99 (d, 1H, *J* = 15.9 Hz), 5.77 (q, 1H, *J* = 6.9 Hz), 5.45 - 5.39 (m, 1H), 4.95 (dd, 1H, *J* = 8.4, 12.2 Hz), 4.94 (dq, 1H, *J* = 6.2, 9.3 Hz), 4.74 (dd, 2H, *J* = 7.1, 16.1 Hz), 4.05 (t, 1H, *J* = 8.7 Hz), 3.78 - 3.72 (m, 1H), 3.65 - 3.58 (m, 1H), 3.53 - 3.50 (m, 2H), 3.38 - 3.26 (m, 1H), 3.36 (s, 3H), 2.82 (dd, 1H, *J* = 8.2, 17.5 Hz), 2.66 - 2.53 (m, 2H), 1.48 (d, 3H, *J* = 6.7 Hz), 1.36 (d, 3H, *J* = 6.2 Hz), 1.30 (d, 3H, *J* = 6.3 Hz); ^13^C-NMR (CDCl_3_, 125 MHz) δ 169.6, 167.8, 164.0, 158.3, 145.3, 125.3, 93.8, 77.6, 76.4, 71.5, 71.4, 68.1, 67.4, 66.5, 58.9, 40.9, 39.0, 19.9, 19.1, 17.6; IR (neat) ν _max_ 2925, 2854, 1742, 1455, 1374, 1252, 1192, 1104, 1051 cm^−1^; LRMS (FAB) *m/z* 444 (M + H^+^); HRMS (FAB) Calcd for C_20_H_30_NO_10_ (M + H^+^): 444.1870, Found 444.1854.

*Macrosphelide B* (**1**)**: **To a solution of MEM protected macrosphelide B [21] (1.6 mg, 3.7 μmol) in CH_2_Cl_2_ (0.3 mL) was added trifluoroacetic acid (0.3 mL) at ambient temperature. After stirring for 3 h, the reaction mixture was concentrated *in vacuo* and purified by flash column chromatography on silica gel (EtOAc-*n-*hexane = 1:2 → 1:1) to afford 1.2 mg (94%) of macrosphelide B (**2**) as a colorless oil: [α]_D_^20^ +10.8 (c 0.065, MeOH); ^1^H-NMR (CDCl_3_, 300 MHz) δ 6.96 (d, 1H, *J* = 15.7 Hz), 6.85 (dd, 1H, *J* = 3.4, 15.5 Hz), 6.68 (d, 1H, *J* = 15.5 Hz), 6.02 (dd, 1H, *J* = 2.0, 15.5 Hz), 5.43 - 5.36 (m, 1H), 5.04 - 4.95 (m, 2H), 4.25 (bs, 1H), 2.76 (dd, 1H, *J* = 10.9, 16.1 Hz), 2.55 (dd, 1H, *J* = 2.3, 16.1 Hz), 1.43 (d, 3H, *J* = 6.6 Hz), 1.37 (d, 3H, *J* = 7.1 Hz), 1.29 (d, 3H, *J* = 6.2 Hz); ^13^C-NMR (CDCl_3_, 75 MHz) δ 196.2, 170.3, 165.4, 164.1, 144.1, 132.5, 132.0, 122.5, 77.2, 75.8, 74.8, 40.5, 19.8, 17.9, 16.1; IR (neat) ν _max_ 3464, 2924, 1725, 1539, 1455, 1265, 1182, 1057 cm^−1^; LRMS (FAB) *m/z* 341 (M + H^+^); HRMS (FAB) Calcd for C_16_H_21_O_8_ (M + H^+^): 341.1236, Found 341.1229.

## 4. Conclusions

We have investigated the catalyst-assisted stereocontrol of INOC in connection with the synthesis of MSPB. Although most of the cycloaddition conditions didn’t provide excellent selectivities, it was notable that PTC-assisted INOC exhibited reverse selectivity. This unprecedented discovery was applied for synthesis of the MSP family as well as other natural polyketides. Further studies on mechanism, increase of facial selectivity and synthetic applications are currently ongoing. 
